# 5-Isopropyl-2-methyl-3-phenyl­sulfonyl-1-benzofuran

**DOI:** 10.1107/S1600536808017224

**Published:** 2008-06-13

**Authors:** Hong Dae Choi, Pil Ja Seo, Byeng Wha Son, Uk Lee

**Affiliations:** a Department of Chemistry, Dongeui University, San 24 Kaya-dong, Busanjin-gu, Busan 614-714, Republic of Korea; b Department of Chemistry, Pukyong National University, 599-1 Daeyeon 3-dong, Nam-gu, Busan 608-737, Republic of Korea

## Abstract

The title compound, C_18_H_18_O_3_S, was prepared by the oxidation of 5-isopropyl-2-methyl-3-phenyl­sulfanyl-1-benzofuran with 3-chloro­peroxy­benzoic acid. The phenyl ring makes a dihedral angle of 79.37 (6)° with the plane of the benzofuran fragment. The crystal structure is stabilized by aromatic π–π stacking inter­actions between the benzene and furan rings of neighbouring mol­ecules [centroid–centroid distance = 3.762 (3) Å]. In addition, the stacked mol­ecules exhibit C—H⋯π and intra­molecular C—H⋯O inter­actions.

## Related literature

For the crystal structures of similar 2-methyl-3-phenyl­sulfonyl-1-benzofuran compounds, see: Choi *et al.* (2008*a* ▶,*b* ▶).
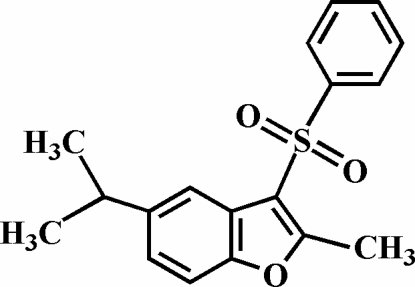


## Experimental

### 

#### Crystal data

C_18_H_18_O_3_S
*M* *_r_* = 314.38
Monoclinic, 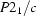

*a* = 10.736 (1) Å
*b* = 13.024 (1) Å
*c* = 11.729 (1) Å
β = 99.655 (2)°
*V* = 1616.8 (2) Å^3^
*Z* = 4
Mo *K*α radiation
μ = 0.21 mm^−1^
*T* = 173 (2) K
0.60 × 0.40 × 0.40 mm

#### Data collection

Bruker SMART CCD diffractometer
Absorption correction: none
9705 measured reflections
3505 independent reflections
3008 reflections with *I* > 2σ(*I*)
*R*_int_ = 0.066

#### Refinement

*R*[*F* ^2^ > 2σ(*F* ^2^)] = 0.049
*wR*(*F* ^2^) = 0.127
*S* = 0.98
3505 reflections
200 parameters
H-atom parameters constrained
Δρ_max_ = 0.25 e Å^−3^
Δρ_min_ = −0.53 e Å^−3^

### 

Data collection: *SMART* (Bruker, 2001 ▶); cell refinement: *SAINT* (Bruker, 2001 ▶); data reduction: *SAINT*; program(s) used to solve structure: *SHELXS97* (Sheldrick, 2008 ▶); program(s) used to refine structure: *SHELXL97* (Sheldrick, 2008 ▶); molecular graphics: *ORTEP-3* (Farrugia, 1997 ▶) and *DIAMOND* (Brandenburg, 1998 ▶); software used to prepare material for publication: *SHELXL97*.

## Supplementary Material

Crystal structure: contains datablocks global, I. DOI: 10.1107/S1600536808017224/rt2021sup1.cif

Structure factors: contains datablocks I. DOI: 10.1107/S1600536808017224/rt2021Isup2.hkl

Additional supplementary materials:  crystallographic information; 3D view; checkCIF report

## Figures and Tables

**Table 1:** Hydrogen-bond geometry (Å, °)

*D*—H⋯*A*	*D*—H	H⋯*A*	*D*⋯*A*	*D*—H⋯*A*
C6—H6⋯*Cg*3^i^	0.95	2.65	3.516 (3)	152
C18—H18*A*⋯O2	0.98	2.39	3.119 (3)	131
C10—H10⋯O2	0.95	2.58	2.938 (2)	103

Symmetry code: (i) 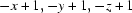. *Cg*3 is the centroid of the C9—C14 phenyl ring.

**Fig. 1.**
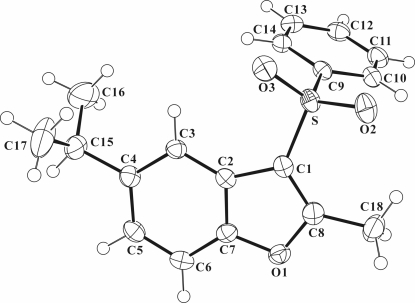
The molecular structure of the title compound, showing displacement ellipsoides drawn at the 50% probability level.

**Fig. 2.**
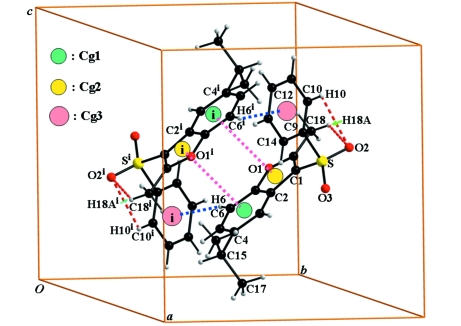
π—π, C—H···π and C—H···O interactions (dotted lines) in the title compound. Cg denotes the ring centroids. [Symmetry code: (i) -x + 1, -y + 1, -z + 1.]

## supplementary crystallographic information

### Comment

As a part of our ongoing studies on the synthesis and structure of 2-methyl-3-phenylsulfonyl-1-benzofuran analogues, the crystal structure of 2,5,7-trimethyl-3-phenylsulfonyl-1-benzofuran (Choi *et al.*, 2008*a*) and 2,5-dimethyl-3-phenylsulfonyl-1-benzofuran (Choi *et al.*, 2008*b*) have been described in the literature. Here we report the crystal structure of the title compound, 5-isopropyl-2-methyl-3-phenylsulfonyl-1-benzofuran (Fig. 1).

The benzofuran unit is essentially planar, with a mean deviation of 0.01° from the least-squares plane defined by the nine constituent atoms. The phenyl ring (C9—C14) makes a dihedral angle of 79.37 (6)° with the plane of the benzofuran fragment. The crystal packing (Fig. 2) is stabilized by aromatic π—π stacking interactions between the benzene ring and the furan ring of neighbouring molecules. The *Cg*1···*Cg*2^i^ distance is 3.762 (3) Å (*Cg*1 and *Cg*2 are the centroids of the C2—C7 benzene ring and the O1/C8/C1/C2/C7 furan ring, respectively, symmetry code as in Fig. 2). The molecular packing (Fig. 2) is further stabilized by the C—H···π interactions between a benzene H atom of the benzofuran system and the phenyl ring of the phenylsulfonyl substitutent, with a C6—H6···*Cg*3^i^ separation of 2.65 Å (Fig. 2 and Table 1; *Cg*3 is the centroid of the C9—C14 phenyl ring, symmetry code as in Fig. 2). Additionally, intramolecular C—H···O interactions in the structure were observed (Fig. 2 and Table 1; symmetry code as in Fig. 2).

### Experimental

3-Chloroperoxybenzoic acid (77%, 471 mg, 2.1 mmol) was added in small portions to a stirred solution of 5-isopropyl-2-methyl-3-phenylsulfanyl-1-benzofuran (282 mg, 1.0 mmol) in dichloromethane (30 ml) at 273 K. After being stirred for 4 h at room temperature, the mixture was washed with saturated sodium bicarbonate solution and the organic layer was separated, dried over magnesium sulfate, filtered and concentrated in vacuum. The residue was purified by column chromatography (hexane-ethyl acetate, 2:1 *v*/*v*) to afford the title compound as a colorless solid [yield 81%, m.p. 386–387 K; *R*_f_ = 0.79 (hexane-ethyl acetate, 1:1 *v*/*v*)]. Single crystals suitable for X-ray diffraction were prepared by evaporation of a solution of the title compound in acetone at room temperature. Spectroscopic analysis: ^1^H NMR (CDCl_3_, 400 MHz) δ 1.28 (d, J = 6.96 Hz, 6H), 2.79 (s, 3H), 2.91–3.07 (m, 1H), 7.18 (dd, J = 8.44 Hz and 1.48 Hz, 1H), 7.33 (d, J = 8.40 Hz, 1H), 7.45–7.57 (m, 3H), 7.69 (s, 1H), 8.01 (dd, J = 6.96 Hz and 1.84 Hz, 2H); EI—MS 314 [*M*^+^].

### Refinement

All H atoms were positioned geometrically and refined using a riding model, with C—H = 0.95 Å for aromatic H atoms, 1.00 Å for methine H atom and 0.98 Å for methyl H atoms, respectively, and with *U*_iso_(H) = 1.2Ueq(C) for aromatic and methine, and 1.5Ueq(C) for methyl H atoms.

### Crystal data

**Table:**

C_18_H_18_O_3_S	*F*_000_ = 664
*M**_r_* = 314.38	*D*_x_ = 1.292 Mg m^−^^3^
Monoclinic, *P*2_1_/*c*	Melting point = 386–387 K
Hall symbol: -p 2ybc	Mo *K*α radiation λ = 0.71073 Å
*a* = 10.736 (1) Å	Cell parameters from 5849 reflections
*b* = 13.024 (1) Å	θ = 2.4–28.1º
*c* = 11.729 (1) Å	µ = 0.21 mm^−^^1^
β = 99.655 (2)º	*T* = 173 (2) K
*V* = 1616.8 (2) Å^3^	Block, colorless
*Z* = 4	0.60 × 0.40 × 0.40 mm

### Data collection

**Table:**

Bruker SMART CCD diffractometer	3505 independent reflections
Radiation source: fine-focus sealed tube	3008 reflections with *I* > 2σ(*I*)
Monochromator: graphite	*R*_int_ = 0.066
Detector resolution: 10.0 pixels mm^-1^	θ_max_ = 27.0º
*T* = 173(2) K	θ_min_ = 2.5º
φ and ω scans	*h* = −13→13
Absorption correction: none	*k* = −15→16
9705 measured reflections	*l* = −13→14

### Refinement

**Table:**

Refinement on *F*^2^	Secondary atom site location: difference Fourier map
Least-squares matrix: full	Hydrogen site location: inferred from neighbouring sites
*R*[*F*^2^ > 2σ(*F*^2^)] = 0.049	H-atom parameters constrained
*wR*(*F*^2^) = 0.127	*w* = 1/[σ^2^(*F*_o_^2^) + (0.061*P*)^2^ + 1.3518*P*] where *P* = (*F*_o_^2^ + 2*F*_c_^2^)/3
*S* = 0.98	(Δ/σ)_max_ = 0.001
3505 reflections	Δρ_max_ = 0.25 e Å^−^^3^
200 parameters	Δρ_min_ = −0.53 e Å^−^^3^
Primary atom site location: structure-invariant direct methods	Extinction correction: none

### Special details

**Table:**

Geometry. All e.s.d.'s (except the e.s.d. in the dihedral angle between two l.s. planes) are estimated using the full covariance matrix. The cell e.s.d.'s are taken into account individually in the estimation of e.s.d.'s in distances, angles and torsion angles; correlations between e.s.d.'s in cell parameters are only used when they are defined by crystal symmetry. An approximate (isotropic) treatment of cell e.s.d.'s is used for estimating e.s.d.'s involving l.s. planes.
Refinement. Refinement of *F*^2^ against ALL reflections. The weighted *R*-factor *wR* and goodness of fit *S* are based on *F*^2^, conventional *R*-factors *R* are based on *F*, with *F* set to zero for negative *F*^2^. The threshold expression of *F*^2^ > σ(*F*^2^) is used only for calculating *R*-factors(gt) *etc*. and is not relevant to the choice of reflections for refinement. *R*-factors based on *F*^2^ are statistically about twice as large as those based on *F*, and *R*- factors based on ALL data will be even larger.

### Fractional atomic coordinates and isotropic or equivalent isotropic displacement parameters (Å^2^)

**Table:**

	*x*	*y*	*z*	*U*_iso_*/*U*_eq_	
S	0.83554 (4)	0.69176 (3)	0.55320 (4)	0.02524 (15)	
O1	0.46657 (13)	0.66684 (10)	0.47014 (12)	0.0288 (3)	
O2	0.84305 (15)	0.79303 (10)	0.60360 (13)	0.0360 (4)	
O3	0.90954 (14)	0.66788 (11)	0.46512 (12)	0.0328 (3)	
C1	0.67826 (18)	0.66590 (13)	0.49602 (15)	0.0239 (4)	
C2	0.63435 (17)	0.58971 (13)	0.40821 (15)	0.0224 (4)	
C3	0.68980 (17)	0.52023 (13)	0.34157 (16)	0.0247 (4)	
H3	0.7791	0.5153	0.3493	0.030*	
C4	0.61150 (18)	0.45785 (14)	0.26310 (17)	0.0273 (4)	
C5	0.47968 (19)	0.46738 (15)	0.25280 (17)	0.0294 (4)	
H5	0.4275	0.4251	0.1986	0.035*	
C6	0.42262 (18)	0.53572 (14)	0.31820 (17)	0.0285 (4)	
H6	0.3334	0.5417	0.3101	0.034*	
C7	0.50303 (17)	0.59463 (13)	0.39592 (15)	0.0241 (4)	
C8	0.5751 (2)	0.70820 (14)	0.53031 (17)	0.0285 (4)	
C9	0.87264 (17)	0.60205 (13)	0.66639 (15)	0.0231 (4)	
C10	0.85507 (18)	0.62898 (15)	0.77698 (16)	0.0287 (4)	
H10	0.8269	0.6959	0.7924	0.034*	
C11	0.8793 (2)	0.55655 (17)	0.86501 (17)	0.0330 (4)	
H11	0.8657	0.5732	0.9408	0.040*	
C12	0.92341 (19)	0.45979 (16)	0.84182 (18)	0.0327 (4)	
H12	0.9401	0.4105	0.9022	0.039*	
C13	0.94327 (18)	0.43436 (15)	0.73172 (17)	0.0300 (4)	
H13	0.9752	0.3684	0.7172	0.036*	
C14	0.91658 (17)	0.50522 (14)	0.64226 (16)	0.0255 (4)	
H14	0.9281	0.4879	0.5661	0.031*	
C15	0.6668 (2)	0.38118 (17)	0.1882 (2)	0.0391 (5)	
H15	0.5962	0.3356	0.1517	0.047*	
C16	0.7664 (3)	0.3128 (2)	0.2585 (3)	0.0692 (9)	
H16A	0.7283	0.2745	0.3158	0.083*	
H16B	0.8356	0.3554	0.2982	0.083*	
H16C	0.7993	0.2645	0.2068	0.083*	
C17	0.7186 (3)	0.4350 (2)	0.0905 (3)	0.0668 (9)	
H17A	0.7851	0.4833	0.1234	0.080*	
H17B	0.6503	0.4725	0.0420	0.080*	
H17C	0.7538	0.3839	0.0435	0.080*	
C18	0.5557 (2)	0.78612 (17)	0.6181 (2)	0.0409 (5)	
H18A	0.6374	0.8155	0.6525	0.061*	
H18B	0.5166	0.7536	0.6786	0.061*	
H18C	0.5004	0.8407	0.5810	0.061*	

### Atomic displacement parameters (Å^2^)

**Table:**

	*U*^11^	*U*^22^	*U*^33^	*U*^12^	*U*^13^	*U*^23^
S	0.0298 (3)	0.0207 (2)	0.0246 (2)	−0.00658 (17)	0.00273 (18)	−0.00024 (16)
O1	0.0279 (7)	0.0279 (7)	0.0305 (7)	0.0059 (5)	0.0044 (5)	0.0014 (5)
O2	0.0483 (9)	0.0211 (7)	0.0376 (8)	−0.0101 (6)	0.0040 (7)	−0.0042 (6)
O3	0.0331 (8)	0.0362 (8)	0.0300 (7)	−0.0074 (6)	0.0079 (6)	0.0017 (6)
C1	0.0303 (9)	0.0194 (8)	0.0213 (8)	−0.0009 (7)	0.0017 (7)	0.0027 (6)
C2	0.0253 (9)	0.0193 (8)	0.0218 (8)	−0.0015 (7)	0.0013 (7)	0.0044 (6)
C3	0.0221 (8)	0.0225 (8)	0.0290 (9)	−0.0016 (7)	0.0025 (7)	−0.0006 (7)
C4	0.0283 (10)	0.0225 (9)	0.0305 (10)	−0.0037 (7)	0.0036 (8)	−0.0014 (7)
C5	0.0286 (10)	0.0273 (9)	0.0303 (10)	−0.0069 (8)	−0.0007 (8)	0.0002 (7)
C6	0.0216 (9)	0.0305 (9)	0.0322 (10)	−0.0010 (7)	0.0007 (8)	0.0058 (8)
C7	0.0262 (9)	0.0224 (8)	0.0237 (9)	0.0042 (7)	0.0040 (7)	0.0062 (7)
C8	0.0355 (10)	0.0223 (9)	0.0268 (9)	0.0028 (7)	0.0029 (8)	0.0039 (7)
C9	0.0216 (8)	0.0241 (8)	0.0228 (9)	−0.0049 (7)	0.0018 (7)	0.0001 (7)
C10	0.0284 (10)	0.0299 (9)	0.0261 (9)	−0.0010 (8)	−0.0004 (7)	−0.0044 (7)
C11	0.0319 (10)	0.0440 (11)	0.0212 (9)	0.0011 (9)	−0.0012 (8)	−0.0002 (8)
C12	0.0284 (10)	0.0388 (11)	0.0289 (10)	0.0025 (8)	−0.0015 (8)	0.0076 (8)
C13	0.0231 (9)	0.0305 (10)	0.0359 (10)	0.0026 (7)	0.0039 (8)	0.0028 (8)
C14	0.0217 (8)	0.0286 (9)	0.0266 (9)	−0.0023 (7)	0.0048 (7)	−0.0018 (7)
C15	0.0326 (11)	0.0333 (11)	0.0517 (13)	−0.0065 (8)	0.0078 (10)	−0.0198 (9)
C16	0.0556 (17)	0.0461 (15)	0.101 (2)	0.0171 (13)	−0.0013 (17)	−0.0282 (15)
C17	0.080 (2)	0.0640 (18)	0.0664 (18)	−0.0203 (15)	0.0404 (16)	−0.0332 (15)
C18	0.0482 (13)	0.0337 (11)	0.0420 (12)	0.0074 (10)	0.0111 (10)	−0.0087 (9)

### Geometric parameters (Å, °)

**Table:**

S—O3	1.439 (2)	C10—C11	1.390 (3)
S—O2	1.442 (1)	C10—H10	0.9500
S—C1	1.742 (2)	C11—C12	1.389 (3)
S—C9	1.763 (2)	C11—H11	0.9500
O1—C8	1.367 (2)	C12—C13	1.384 (3)
O1—C7	1.382 (2)	C12—H12	0.9500
C1—C8	1.357 (3)	C13—C14	1.391 (3)
C1—C2	1.451 (2)	C13—H13	0.9500
C2—C7	1.394 (3)	C14—H14	0.9500
C2—C3	1.393 (3)	C15—C16	1.524 (4)
C3—C4	1.398 (3)	C15—C17	1.525 (4)
C3—H3	0.9500	C15—H15	1.0000
C4—C5	1.406 (3)	C16—H16A	0.9800
C4—C15	1.515 (3)	C16—H16B	0.9800
C5—C6	1.383 (3)	C16—H16C	0.9800
C5—H5	0.9500	C17—H17A	0.9800
C6—C7	1.379 (3)	C17—H17B	0.9800
C6—H6	0.9500	C17—H17C	0.9800
C8—C18	1.485 (3)	C18—H18A	0.9800
C9—C10	1.387 (3)	C18—H18B	0.9800
C9—C14	1.392 (3)	C18—H18C	0.9800
			
O3—S—O2	119.50 (9)	C10—C11—C12	119.8 (2)
O3—S—C1	107.34 (9)	C10—C11—H11	120.1
O2—S—C1	108.58 (9)	C12—C11—H11	120.1
O3—S—C9	108.37 (9)	C13—C12—C11	120.7 (2)
O2—S—C9	107.90 (9)	C13—C12—H12	119.6
C1—S—C9	104.11 (8)	C11—C12—H12	119.6
C8—O1—C7	106.6 (2)	C12—C13—C14	120.1 (2)
C8—C1—C2	107.7 (2)	C12—C13—H13	120.0
C8—C1—S	126.5 (2)	C14—C13—H13	120.0
C2—C1—S	125.7 (1)	C13—C14—C9	118.8 (2)
C7—C2—C3	119.5 (2)	C13—C14—H14	120.6
C7—C2—C1	104.2 (2)	C9—C14—H14	120.6
C3—C2—C1	136.4 (2)	C4—C15—C16	112.2 (2)
C2—C3—C4	118.7 (2)	C4—C15—C17	111.1 (2)
C2—C3—H3	120.6	C16—C15—C17	111.4 (3)
C4—C3—H3	120.6	C4—C15—H15	107.3
C5—C4—C3	119.4 (2)	C16—C15—H15	107.3
C5—C4—C15	119.7 (2)	C17—C15—H15	107.3
C3—C4—C15	120.9 (2)	C15—C16—H16A	109.5
C6—C5—C4	122.8 (2)	C15—C16—H16B	109.5
C6—C5—H5	118.6	H16A—C16—H16B	109.5
C4—C5—H5	118.6	C15—C16—H16C	109.5
C5—C6—C7	116.0 (2)	H16A—C16—H16C	109.5
C5—C6—H6	122.0	H16B—C16—H16C	109.5
C7—C6—H6	122.0	C15—C17—H17A	109.5
C6—C7—O1	125.7 (2)	C15—C17—H17B	109.5
C6—C7—C2	123.6 (2)	H17A—C17—H17B	109.5
O1—C7—C2	110.8 (2)	C15—C17—H17C	109.5
C1—C8—O1	110.8 (2)	H17A—C17—H17C	109.5
C1—C8—C18	134.4 (2)	H17B—C17—H17C	109.5
O1—C8—C18	114.9 (2)	C8—C18—H18A	109.5
C10—C9—C14	121.6 (2)	C8—C18—H18B	109.5
C10—C9—S	119.2 (1)	H18A—C18—H18B	109.5
C14—C9—S	119.3 (1)	C8—C18—H18C	109.5
C9—C10—C11	119.0 (2)	H18A—C18—H18C	109.5
C9—C10—H10	120.5	H18B—C18—H18C	109.5
C11—C10—H10	120.5		
			
O3—S—C1—C8	−155.70 (16)	C2—C1—C8—O1	−1.1 (2)
O2—S—C1—C8	−25.23 (19)	S—C1—C8—O1	−177.24 (12)
C9—S—C1—C8	89.53 (18)	C2—C1—C8—C18	177.6 (2)
O3—S—C1—C2	28.81 (17)	S—C1—C8—C18	1.5 (3)
O2—S—C1—C2	159.29 (15)	C7—O1—C8—C1	0.7 (2)
C9—S—C1—C2	−85.95 (16)	C7—O1—C8—C18	−178.25 (16)
C8—C1—C2—C7	0.97 (19)	O3—S—C9—C10	154.95 (15)
S—C1—C2—C7	177.16 (13)	O2—S—C9—C10	24.22 (18)
C8—C1—C2—C3	−178.7 (2)	C1—S—C9—C10	−91.01 (16)
S—C1—C2—C3	−2.5 (3)	O3—S—C9—C14	−26.26 (17)
C7—C2—C3—C4	0.6 (3)	O2—S—C9—C14	−156.99 (14)
C1—C2—C3—C4	−179.76 (19)	C1—S—C9—C14	87.77 (16)
C2—C3—C4—C5	0.5 (3)	C14—C9—C10—C11	−1.5 (3)
C2—C3—C4—C15	179.81 (18)	S—C9—C10—C11	177.28 (15)
C3—C4—C5—C6	−0.7 (3)	C9—C10—C11—C12	1.6 (3)
C15—C4—C5—C6	−179.93 (19)	C10—C11—C12—C13	−0.2 (3)
C4—C5—C6—C7	−0.4 (3)	C11—C12—C13—C14	−1.3 (3)
C5—C6—C7—O1	−179.49 (16)	C12—C13—C14—C9	1.4 (3)
C5—C6—C7—C2	1.6 (3)	C10—C9—C14—C13	0.0 (3)
C8—O1—C7—C6	−179.09 (17)	S—C9—C14—C13	−178.78 (14)
C8—O1—C7—C2	−0.08 (19)	C5—C4—C15—C16	−129.9 (2)
C3—C2—C7—C6	−1.8 (3)	C3—C4—C15—C16	50.8 (3)
C1—C2—C7—C6	178.49 (17)	C5—C4—C15—C17	104.7 (2)
C3—C2—C7—O1	179.20 (15)	C3—C4—C15—C17	−74.6 (3)
C1—C2—C7—O1	−0.54 (19)		

### Hydrogen-bond geometry (Å, °)

**Table:**

*D*—H···*A*	*D*—H	H···*A*	*D*···*A*	*D*—H···*A*
C6—H6···Cg3^i^	0.95	2.65	3.516 (3)	152
C18—H18A···O2	0.98	2.39	3.119 (3)	131
C10—H10···O2	0.95	2.58	2.938 (2)	103

Symmetry codes: (i) −*x*+1, −*y*+1, −*z*+1.

## References

[bb1] Brandenburg, K. (1998). *DIAMOND* Crystal Impact GbR, Bonn, Germany.

[bb2] Bruker (2001). *SAINT* and *SMART* Bruker AXS Inc., Madison, Wisconsin, USA.

[bb3] Choi, H. D., Seo, P. J., Son, B. W. & Lee, U. (2008*a*). *Acta Cryst.* E**64**, o794. 10.1107/S1600536808008477 PMC2961306 21202286

[bb4] Choi, H. D., Seo, P. J., Son, B. W. & Lee, U. (2008*b*). *Acta Cryst.* E**64**, o850. 10.1107/S1600536808009811 PMC2961094 21202338

[bb5] Farrugia, L. J. (1997). *J. Appl. Cryst.* **30**, 565.

[bb6] Sheldrick, G. M. (2008). *Acta Cryst.* A**64**, 112–122. 10.1107/S0108767307043930 18156677

